# Zoledronic Acid as an Adjuvant Therapy in Patients with Breast Cancer: A Systematic Review and Meta-Analysis

**DOI:** 10.1371/journal.pone.0040783

**Published:** 2012-07-26

**Authors:** Wei-Wei Huang, Cheng Huang, Jian Liu, Hong-Yu Zheng, Lin Lin

**Affiliations:** Department of Medical Oncology, Fujian Provincial Cancer Hospital, the Teaching Hospital of Fujian Medical University, Fuzhou, China; Istituto di Ricerche Farmacologiche Mario Negri, Italy

## Abstract

**Background:**

Zoledronic acid is widely used as adjuvant chemotherapy for the treatment of breast cancer. However, previous trials reported inconsistent findings regarding their clinical efficacy and safety. We carried out a comprehensive systematic review and meta-analysis to evaluate the effects of zoledronic acid on disease-free survival (DFS), overall survival (OS), and drug-related toxicities.

**Methodology and Principal Findings:**

We systematically searched Medline, EmBase, the Cochrane Central Register of Controlled Trials, reference lists of articles and proceedings of major meetings for relevant literatures with a time limit of Dec. 1, 2011. Randomized controlled trials evaluated the effects of zoledronic acid on OS, DFS, and RFS compared with control were eligible for inclusion in our research. Of 175 identified studies, we collected data from 7 randomized controlled trials of zoledronic acid that had OS, DFS, and RFS reported as one of the endpoint. Overall, we noted that patients receiving zoledronic acid therapy had significant longer OS than the group with non-zoledronic acid therapy (HR, 0.85, 95%CI, 0.73 to 1.00, P = 0.047). Furthermore, zoledronic acid therapy also had a clear effect on frature events (RR, 0.66, 95%CI, 0.52 to 0.84, P<0.001). Subgroup analysis indicated that zoledronic acid therapy showed a great beneficial effect on disease recurrence in patients with early-stage breast cancer, however, it also significantly increased the harm of disease recurrence in patients with advanced breast cancer. Bone pain, neutropenic fever, pyrexia, rash were more frequent in the zoledronic acid therapy group.

**Conclusion/Significance:**

Treatment with zoledronic acid had a clear effect on fracture events, and it might contribute an important role for overall survival.

## Introduction

Breast cancer is the leading cause of premature morbidity and mortality worldwide for women, approximately 800,000 women are diagnosed with breast cancer, and an estimated 65% to 75% of patients with advanced metastases breast cancer will develop bone metastases during the course of their disease[1]–[2]. Over the past few years, many study indicated that bone metastases are common in patients with advanced breast cancer, which resulted in significant skeletal morbidity [3]–[4]. For these patients, zoledronic acid has emerged as a new drug commonly used for the treatment of bone metastases in patients with breast cancer, and evidence showed that zoledronic acid was the most effective in patients with metastases breast cancer [5]–[7].

The goals of prevention for patients with breast cancer are reduce rates of recurrence or death [8]. In patients with advanced breast cancer, metastatic tumor cells in bone may secrete cytokines and growth factors that induce osteoblasts to release receptor activator of nuclear factor RANKL, a key mediator of osteoclast formation, function and survival, which increase in osteoclastic bone resorption lead to the release of bone-derived growth factors that may provide a fertile environment for survival and growth of adjacent cancer cells [9]–[10]. Therefore, targeting bone-cell function provided a potential additional approach to prevent bone metastases as a component of standard adjuvant therapy.

Recently, several large-scale randomized controlled trials of adjuvant zoledronic acid therapy for patients with breast cancer have been completed. A number of these trials indicated that adjuvant therapy had some beneficial effect on overall survival (OS), disease free survival (DFS), and recurrence free survival, whereas others showed that it had limited effects in one or more specific index, and some even found that it could induce drug-related adverse reactions- nephrotoxicity. This led uncertainty over the presence and magnitude of any protective the recurrence or death in patients with advanced metastases breast cancer of zoledronic acid therapy and difficulties in interpretation of the results. To better understand the efficacy of zoledronic acid therapy on patients with breast cancer, data from these recent trials needed to be re-evaluated to formulate a conclusion regarding the efficacy and safety of zoledronic acid therapy. We undertook a comprehensive systematic review and meta-analysis to update the results and resolve the uncertain efficacy and safety of zoledronic acid in women with breast cancer, furthermore, we also provided more detail conclusion for the efficacy of zoledronic acid therapy in some specific subsets.

**Figure 1:**
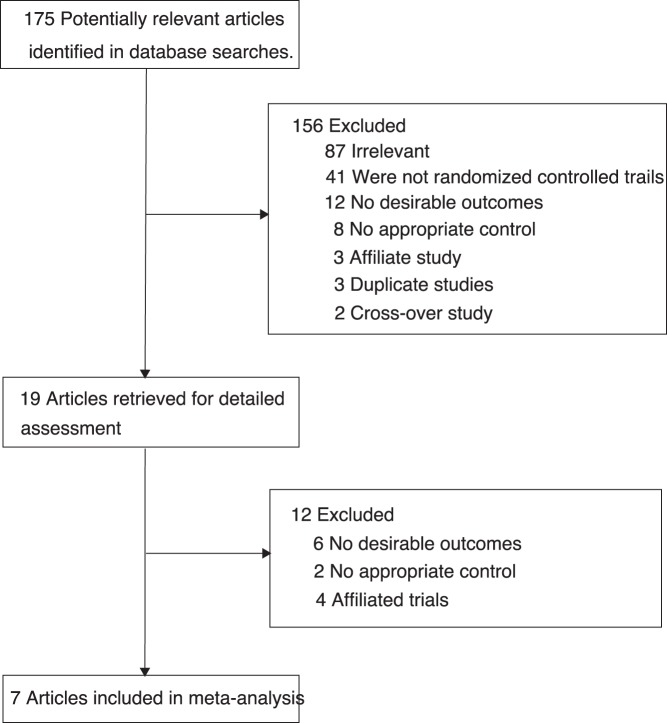
Diagram of the literature search and tria selection process.

## Methods

### Data Sources, Search Strategy, and Selection Criteria

Randomized controlled trials of zoledronic acid therapy in English-language literature were eligible in our meta-analysis regardless of publication status (published, unpublished, in press, or in progress). Searched process as the following procedure:

1. Electronic searches: We searched PubMed, EmBase, and the Cochrane Central Register of Controlled Trials with a date limit of Dec 20, 2011, with the terms of “zoledronic acid” AND “breast cancer” AND “randomized controlled trials”. All reference lists from reports on non-randomized controlled trials were searched manually for additional eligible studies.
2. Other source: We contacted authors to obtain any possible additional published or unpublished data and searched the proceedings of the annual meeting in the Cochrane Central Register. Furthermore, references were also identified by screening the proceedings of the annual meeting, bibliographies of publications for potentially relevant trials.

We restricted our research to randomized controlled trials, which are less likely to be subject to confounding bias than are observational studies. The literature search was undertaken independently by 2 authors (Cheng Huang and Jian Liu) with a standardized approach, and any disagreement between these 2 authors was settled by primary author (Wei-Wei Huang) until a consensus was reached. Furthermore, identified trials had to report on 1 or more of the following primary or secondary outcomes: overall survival (OS), disease free survival (DFS), recurrence free survival (RFS), and other possible adverse drug-related reaction.

**Table 1:** Design and characteristic of trials included in the systematic review and meta-analysis.

Source	No. of patients	Meanage, y	Inclusion criteria	Intervention	Follow-up(month)	Jadadscore
AT Stopeck [15]	2046	56.5	advanced breast cancer	(1) zoledronic acid 4 mg every4 weeks;(2) denosumab 120 mg	34	3
Z-FAST Study [19]	602	61.2	early-stage breast cancer	(1) immediate zoledronic acid4 mg every 6 month;(2) delayed zoledronic acid4 mg every 6 month	60	4
ABCSG-12 Trial Investigators [16]	1803	44.5	early-stage breast cancer	(1) zoledronic acid 4 mg every6 month(2) non-zoledronic acid therapy	62	4
Rebecca A [17]	120	48.0	locally advanced breast cancer	(1) zoledronic acid 4 mg every3 weeks;(2) non-zoledronic acid therapy	24	2
AZURE Investigators [18]	3360	>18 y	breast cancer with axillary lymph- node metastasis(N1) or a T3–T4 primarytumor.	(1) zoledronic acid every 3 to4 weeks for 6 doses and thenevery 3 to 6 months to complete5 years of treatment.(2) non-zoledronic acid therapy	59	4
ZO-FAST Study [20]	1065	57.5	early breast cancer	(1) immediate zoledronic acid4 mg every 6 month;(2) delayed zoledronic acid4 mg every 6 month	36	4
E-ZO-FAST Study [21]	522	58.0	early-stage breast cancer	(1) immediate zoledronic acid4 mg every 6 month;(2) delayed zoledronic acid4 mg every 6 month	12	3

**Figure 2:**
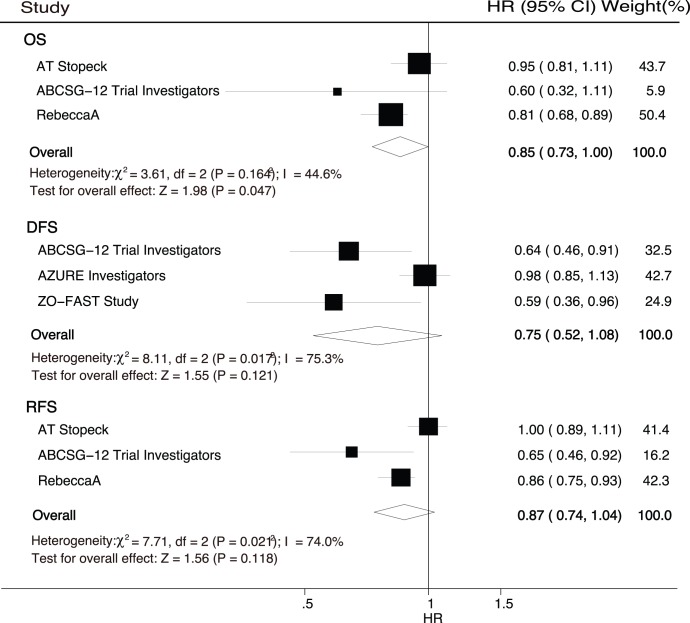
Comparison of OS, DFS, RFS between zoledronic acid therapy and control.

### Data Collection and Quality Assessment

The identified 175 studies were reviewed by 2 authors (Cheng Huang and Jian Liu) independently. Other two investigators (Hong-Yu Zheng, Lin Lin) independently checked each full-text trial for eligibility and extracted and tabulated all relevant data with a standard protocol and reviewed by a third investigators (Wei-Wei Huang). Any discrepancy was settled by group discussion, after which the primary authors (Wei-Wei Huang) made the final decision. Extracted data included: first author or study group’s name, year of publication, number of patients, mean age, sex, study design, regimen details, follow-up, disease status, the hazard ratios (HR) and its 95% confidence interval (95% CI), or the drug-related toxicities. We also attempted to contact the authors to obtain more detail information. Study quality was assessed using the Jadad score [11], which based on randomization, concealment of the treatment allocation, blinding, completeness of follow-up, and the use of intention-to treat analysis.

**Table 2:** Summary of the relative risks of all outcomes assessed.

Outcomes	Included studies	RR and 95% CI	P value	heterogeneity	P value for heterogeneity
Total death	16,18,19–21	0.91 (0.69, 1.20)	0.51	14%	0.33
Disease recurrence	16,18–21	0.82 (0.51, 1.32)	0.41	77%	0.001
Fracture	15,16,18–21	0.66 (0.52, 0.84)	0.0006	0%	0.62
Bone pain	15–17,19–21	1.42 (1.28, 1.57)	<0.0001	0%	0.55
Infection	17,19	1.24 (0.74, 2.09)	0.41	0%	0.42
Neutropenic fever	16,17	3.52 (1.80, 6.87)	0.0002	15%	0.28
Diarrhoea	15,17,19	0.92 (0.78, 1.08)	0.28	0%	0.49
Nausea	15,19–21	1.10 (0.99, 1.22)	0.08	0%	0.92
Constipation	15,19,20	0.99 (0.68, 1.42)	0.94	51%	0.13
Fatigue	15,16,19–21	1.08 (0.98, 1.18)	0.11	0%	0.66
Peripheral edema	16,19–21	1.28 (0.99, 1.65)	0.05	0%	0.42
Pyrexia	15,18–21	2.97 (1.46, 6.04)	0.003	87%	<0.0001
Arthralgia	15,16,19–21	1.09 (0.97, 1.22)	0.16	55%	0.06
Myalgia	19–21	1.11 (0.88, 1.38)	0.38	0%	0.44
Headache	16,19–21	1.11 (0.89, 1.38)	0.36	27%	0.25
Dizziness	16,19,20	0.93 (0.60, 1.42)	0.72	36%	0.21
Depression	16,19–21	0.80 (0.61, 1.03)	0.08	31%	0.23
Insomnia	19,20	1.17 (0.83, 1.65)	0.36	0%	0.98
Anxiety	19,21	0.74 (0.44, 1.25)	0.26	0%	0.82
Cough	19,20	0.73 (0.35, 1.51)	0.39	71%	0.06
Dyspnea	15,19	0.96 (0.65, 1.43)	0.86	41%	0.19
Rash	16,19	1.47 (1.04, 2.07)	0.03	0%	0.85
Hot flush	19–21	0.87 (0.75, 1.01)	0.07	13%	0.32

### Statistical Analysis

Hazard ratios (HR) or relative risk (RR) with its 95% confidence interval (CI) was calculated for outcomes extracted from each trial before data pooling. We used hazard ratios (HR) with its 95%CI for overall survival (OS), disease free survival (DFS), and recurrence free survival (RFS) to evaluate the efficacy of zoledronic acid, and relative risk (RR) with its 95%CI for adverse drug-related reaction to assess the safety of zoledronic acid. We also did a stratified analysis by mean age, number of patients, disease status, control drug, duration of follow-up, or Jadad score. Although the fixed-effect and random-effects models yielded similar conclusions, The statistical estimates of effect were derived using a random-effects model with Mantel-Haenszel statistics, which assumes that the true underlying effect varies among included trials, because of the different characteristic of diseases, intervention regimens, the duration of follow-up that were involved in the original trials. Moreover, many investigators also consider the random-effects model to be a more natural choice than the fixed-effect model in medical decision-making contexts [12]–[13]. Heterogeneity of treatment effects among studies was investigated visually by scatter plot and statistically by the heterogeneity I^2^ statistic [14]. All the reported P values were two-side and P values less than 0.05 were regarded as significant for all the included studies. Statistic analyses were carried out using STATA (version 10.0).

## Results

We identified 175 potential studies from our initial electronic search, and excluded 156 trials after a preliminary review. Nineteen potential trials were identified and then twelve of them were exclude for specific reason listed in Figure 1. Of these, 7 randomized controlled trials met our inclusion criteria. 4 of included trials [15]–[18] evaluating zoledronic acid therapy compared to non-zoledronic acid therapy and the remaining three trials [19]–[21] assessing immediate zoledronic acid therapy compared with delayed zoledronic acid. Of 7 included studies, which consisted of data of 9518 patients with breast cancer. Table 1 summarizes the baseline characteristics of the included studies and their participants. The sample size ranged from 120 to 3360, with a mean of 1360, and the follow-up for patients ranged from 12 to 62 months. The primary outcomes were overall survival (OS) available in 3 trials, disease free survival (DFS) in 3 trials, and recurrence free survival in 3 trials. The quality of the trials was assessed according to the pre-fixed criteria using Jadad score. Overall, of the 7 including randomized controlled trials, four trials scored 4, two scored 3, and one scored 2.

**Table 3:** Subgroup analysis for the effect of zoledronic acid therapy on total death, disease recurrence, and fracture.

	Subgroup		Intervention group	Control group	RR(95% CI)	P value	P value for heterogeneity
Total death	Mean age	>50	12/1077	5/1107	2.14 [0.76, 6.00]	0.15	0.58
		<50	270/2640	311/2642	0.87 [0.75, 1.01]	0.07	0.39
	Number of patients	>1000	263/3104	305/3118	0.86 [0.74, 1.01]	0.06	0.46
		<1000	19/613	11/631	1.54 [0.77, 3.11]	0.22	0.40
	Disease status	Early-stage	28/1976	31/2011	1.23 [0.49, 3.12]	0.66	0.16
		Advanced	254/1741	285/1738	0.89 [0.76, 1.04]	0.14	0.43
	Control drug	Delayed zoledronic acidtherapy	12/1077	5/1107	2.14 [0.76, 6.00]	0.15	0.58
		Non-zoledronic acidtherapy	270/2640	311/2642	0.87 [0.75, 1.01]	0.07	0.39
	Follow-up (month)	>36	268/3405	307/3419	0.87 [0.71, 1.06]	0.17	0.37
		<36	14/312	9/330	1.74 [0.41, 7.43]	0.45	0.23
	Jadad score	4 or 5	268/3405	307/3419	0.87 [0.71, 1.06]	0.17	0.37
		<4	14/312	9/330	1.74 [0.41, 7.43]	0.45	0.23
Diseaserecurrence	Mean age	>50	38/1077	61/1107	0.66 [0.41, 1.06]	0.08	0.28
		<50	173/2580	160/2582	0.95 [0.46, 1.96]	0.88	0.002
	Number of patients	>1000	195/3104	200/3118	0.81 [0.44, 1.47]	0.49	0.0005
		<1000	16/553	21/571	0.84 [0.33, 2.17]	0.72	0.17
	Disease status	Early-stage	77/1976	122/2011	0.64 [0.48, 0.85]	0.002	0.47
		Advanced	134/1681	99/1678	1.35 [1.05, 1.74]	0.02	-
	Control drug	Delayed zoledronic acidtherapy	38/1077	61/1107	0.66 [0.41, 1.06]	0.08	0.28
		Non-zoledronic acidtherapy	173/2580	160/2582	0.95 [0.46, 1.96]	0.88	0.002
	Follow-up (month)	>36	204/3405	216/3419	0.76 [0.45, 1.28]	0.30	0.0007
		<36	7/252	5/270	1.50 [0.48, 4.67]	0.48	-
	Jadad score	4 or 5	204/3405	216/3419	0.76 [0.45, 1.28]	0.30	0.0007
		<4	7/252	5/270	1.50 [0.48, 4.67]	0.48	-
Fracture	Mean age	>50	45/1076	56/1106	0.82 [0.56, 1.21]	0.32	0.71
		<50	66/2585	112/2570	0.58 [0.43, 0.78]	0.0004	0.90
	Number of patients	>1000	92/3109	144/3106	0.64 [0.49, 0.82]	0.0006	0.49
		<1000	19/552	24/570	0.81 [0.45, 1.47]	0.49	0.41
	Disease status	Early-stage	46/1975	58/2010	0.81 [0.56, 1.19]	0.28	0.84
		Advanced	65/1686	110/1666	0.58 [0.43, 0.79]	0.0004	-
	Control drug	Delayed zoledronic acidtherapy	45/1076	56/1106	0.82 [0.56, 1.21]	0.32	0.71
		Non-zoledronic acidtherapy	66/2585	112/2570	0.58 [0.43, 0.78]	0.0004	0.90
	Follow-up (month)	>36	109/3409	163/3406	0.67 [0.53, 0.85]	0.0009	0.50
		<36	2/252	5/270	0.43 [0.08, 2.19]	0.31	-
	Jadad score	4 or 5	109/3409	163/3406	0.67 [0.53, 0.85]	0.0009	0.50
		<4	2/252	5/270	0.43 [0.08, 2.19]	0.31	-

Data for OS were available from 3 trials, including 3969 patients who were recruited in our research on the risk of death. We noted that zoledronic acid therapy was associated with a clinically and statistically significant 15% improvement in OS when compared with the control (HR, 0.85, 95%CI, 0.73 to 1.00, P = 0.047, Figure 2). However, we noted that the pooled RR showed a 9% reduction in the event of total death, and with no evidence showed that zoledronic therapy protected against total death risk (RR, 0.91, 95%CI, 0.69 to 1.20, with unimportant heterogeneity, table 2).

DFS was reported in 3 trials of the seven included studies. Overall, we noted that zoledronic acid therapy had no effect on the risk of DFS as compared with the control (HR, 0.75, 95%CI, 0.52 to 1.08, P = 0.121, Figure 2). Furthermore, although zoledronic acid therapy reduced the risk of disease recurrence by 18%, however, the effect of zoledronic acid on the risk of disease recurrence was not associated with a statistically significant (RR, 0.82, 95%CI, 0.51 to 1.32, Table 2). Although there was some evidence of heterogeneity across the studies included, a sensitivity analysis indicated that the results were not affected by sequential exclusion of any particular trial from all pooled analysis.

The risk of recurrence-free survival (RFS) was reported in 3 trials, after pooling these trials, we observed that no effect of zoledronic acid therapy on the risk of RFS (HR, 0.87, 95%CI, 0.74 to 1.04, P = 0.118, Figure 2). Furthermore, we noted that with zoledronic therapy the risk of fracture was significantly reduced by 34% (RR, 0.66, 95%CI, 0.52 to 0.84, without evidence of heterogeneity of effect, Table 2).

Data concerning drug-related adverse effects were extracted from 7 trials. A summary of drug-related toxicities was shown in Table 2. Overall, we noted that zoledronic acid therapy as compared to control produced an 42% significant increase in the risk of bone pain (RR, 1.42, 95%CI, 1.28 to 1.57), 252% RR increase (RR, 3.52, 95%CI, 1.80 to 6.87) for Neutropenic fever, 197% RR increase (RR, 2.97, 95%CI, 1.46 to 6.04) for Pyrexia, and 47% RR increase (RR, 1.47, 95%CI, 1.04 to 2.07) for rash.

Subgroup analyses were carried out for total death, disease recurrence, and fracture. Overall, we noted that zoledronic acid therapy was associated with a reduction in the risk of disease recurrence, when patients with early-stage breast cancer (RR, 0.64, 95%CI, 0.48 to 0.85), on the contrary, zoledronic acid therapy as compared to control produced an 35% significant increase in the risk of disease recurrence in patients with advanced breast cancer (RR, 1.35, 95%CI, 1.05 to 1.74). Furthermore, zoledronic acid therapy showed a clear effect on fracture events when the mean age of the patients was less than 50, sample size more than 1000, the patients with advanced breast cancer, compared with non-zoledronic acid therapy, the follow-up more than 36 months, and Jadad score 4 or 5. No other significant differences were identified between the efficacy of zoledronic therapy and control, based on additional subset factors (Table 3).

## Discussion

This comprehensive systematic review and meta-analysis included 9518 patients with breast cancer, which with a broad range of baseline characteristics. The pooled HRs for OS indicated that zoledronic acid therapy was associated with significant improvement as compared control. For drug-related effects, we noted that zoledronic acid therapy was associated with a reduction in the risk of fracture event. In addition, zoledronic acid therapy was associated with a significant increased the risk of bone pain, neutropenic fever, pyrexia and rash.

Zoledronic acid, a potent nitrogen-containing bisphos-phonate, has emerged as a new drug commonly used for maintain or increase bone mineral density (BMD) in premenopausal women with early-stage breast cancer with low BMD [22]–[24]. However, the effect of zoledronic acid therapy concerning improvement of breast cancer patient survival remained unclear. Previous research [25] indicated that adjunctive of zoledronic acid to standard therapy could significantly improve DFS and reduces the risk of distant and locoregional recurrence, however, which based on absolute number on patients, which not provided the result based on survival data, furthermore, additional randomized controlled trials have been completed. Therefore, we carried out a systematic review and meta-analysis to explain the possible effect of zoledronic acid therapy on OS, DFS, RFS, total death, disease recurrence, and any possible drug-related adverse reactions.

Previous meta-analysis [25] indicated that zoledronic acid had a clear effect on fracture events. The main findings of our meta-analysis as compared with previous research, which indicated that zoledronic acid therapy was associated with a clinically and statistically improvement in OS, but not DFS and RFS, it also supported the conclusion by Yan et al [25]. Although zoledronic acid had a limit effects on DFS and RFS, however, these results might easily vary.

No significant differences in the relative risk of total death and disease recurrence were detected across a wide baseline characteristic of patients in these included trials. In our research, 4 trials [16], [19]–[21] provided patients with early-stage breast cancer, and other 3 trials [15], [17], [18] provided patients with advanced cancer, in addition, AZURE Investigators trials [18] not only include postmenopausal patients, but also premenopausal women. We therefore did a subgroup analysis to explore any possible variations based on baseline characteristic of patients.

AZURE Investigators trials [18] suggested that no improvement was seen in the rate of disease-free survival, rates of invasive disease free survival and overall survival were similar between the treatment group and control. The ABCSG-12 Study [16] illustrated that addition of zoledronic acid to endocrine therapy, as compared with endocrine therapy alone, resulted in a relative reduction of 36%, nearly one third in the risk of disease progression. Our research suggested that no significant difference in the relative risk of disease recurrence was detected, the reason for this absence of difference could be that in the ABCSG-12 study, patients with early-stage breast cancer started receiving goserelin and endocrine therapy before the initiation of bisphosphonate treatment, in addition, patients with early-stage breast cancer often with a good prognosis, and less than 5% received chemotherapy.

We noted that zoledronic acid therapy had significant longer OS (HR, 0.85, 95%CI, 0.73 to 1.00) than the control group. However, zoledronic acid therapy had a limit effect on total death. The reason for this absence difference could be that difference follow-up contributed inconsistent conclusion. Although only 3 trials provided survival data on overall survival, the pooled analysis on overall survival was more exactly than total death.

Subgroup analyses were performed based on mean age, number of patients, disease status, control drug, follow-up, and Jadad score. Overall, we noted that zoledronic acid was significantly reduced the risk of disease recurrence in patients with early-stage breast cancer, however, zoledronic acid therapy was significantly increased the risk of disease recurrence in patients with advanced breast cancer. The reason could be that the patients with advanced breast cancer had a high recurrence rate, and less trial provided the result of disease recurrence, which caused such conclusion easily variable. Furthermore, we noted that zoledronic acid therapy showed a clear effect on fracture events, and subgroup analysis also supported this conclusion when the mean age of the patients less than 50, the number of patients more than 1000, the patients with advanced breast cancer, compared with non-zoledronic acid therapy, the follow-up more than 36 months, and the Jadad score 4 or 5. The reason could be that bone metastases often occurred in 65% to 75% of patients with advanced breast cancer, and most bone metastases have an osteolytic appearance on radiographs, and zoledronic acid has already demonstrated favorable efficacy and safety for the treatment of skeletal complications from bone lesions [26]–[28].

According to our research, we also detected that zoledronic acid significantly increase in the risk of bone pain, neutropenic fever, pyrexia, and rash. These adverse events were consistent with already known drug-safety profiles. Other important factor could be that some of these included studies reported nonstandard adverse effects caused less trial provided adverse events in some special effect.

The limitations of our research are as follows: (i) The conclusion of overall survival and total death contributed inconsistent results, although overall survival provided more exactly result, however, only 3 trials reported such information. (ii) Although subgroup analysis suggested that zoledronic acid was significantly reduced the risk of disease recurrence in patients with early-stage breast cancer, and significantly increased the risk of disease recurrence in patients with advanced breast cancer. However, these results may be variable because of the small number of trials that were included in such subset. (iii) Inherent assumptions made for any meta-analysis, because the analysis used pooled data either published or provided by individual study authors, and individual patient data or original data were unavailable, which restricted us doing more detailed relevant analysis and obtaining more comprehensive results.

In conclusion, the findings of this study indicated that the zoledronic acid had a clear effect on fracture events. Furthermore, it might contribute an important role on overall survival. In future research, it is important to focus on patients with early-stage breast cancer or advanced breast cancer for explored the difference between different disease statuses. We suggest that the ongoing trials be improved in the following ways: (i) The adverse effects in clinical trials should be recorded and reported normatively, so that the side-effects of any treatment can be evaluated in future trials. ii) The role of treatment duration and dosage should be investigated in more detail to explore optimal dose and duration of treatment. iii) survival data, such as OS, DFS, should be recorded in more detail.
